# Oxidative Stress-Responsive MicroRNAs in Heart Injury

**DOI:** 10.3390/ijms21010358

**Published:** 2020-01-05

**Authors:** Branislav Kura, Barbara Szeiffova Bacova, Barbora Kalocayova, Matus Sykora, Jan Slezak

**Affiliations:** 1 Centre of Experimental Medicine, Institute for Heart Research, Slovak Academy of Sciences, 841 04 Bratislava, Slovakia; branislav.kura@savba.sk (B.K.); barbara.bacova@savba.sk (B.S.B.); barbora.kalocayova@savba.sk (B.K.); matus.sykora@savba.sk (M.S.); 2 Department of Animal Physiology and Ethology, Faculty of Natural Sciences, Comenius University, 842 15 Bratislava, Slovakia

**Keywords:** cardiovascular diseases, ischemia/reperfusion injury, miRNA, oxidative stress, transplantation

## Abstract

Reactive oxygen species (ROS) are important molecules in the living organisms as a part of many signaling pathways. However, if overproduced, they also play a significant role in the development of cardiovascular diseases, such as arrhythmia, cardiomyopathy, ischemia/reperfusion injury (e.g., myocardial infarction and heart transplantation), and heart failure. As a result of oxidative stress action, apoptosis, hypertrophy, and fibrosis may occur. MicroRNAs (miRNAs) represent important endogenous nucleotides that regulate many biological processes, including those involved in heart damage caused by oxidative stress. Oxidative stress can alter the expression level of many miRNAs. These changes in miRNA expression occur mainly via modulation of nuclear factor erythroid 2-related factor 2 (Nrf2), sirtuins, calcineurin/nuclear factor of activated T cell (NFAT), or nuclear factor kappa B (NF-κB) pathways. Up until now, several circulating miRNAs have been reported to be potential biomarkers of ROS-related cardiac diseases, including myocardial infarction, hypertrophy, ischemia/reperfusion, and heart failure, such as miRNA-499, miRNA-199, miRNA-21, miRNA-144, miRNA-208a, miRNA-34a, etc. On the other hand, a lot of studies are aimed at using miRNAs for therapeutic purposes. This review points to the need for studying the role of redox-sensitive miRNAs, to identify more effective biomarkers and develop better therapeutic targets for oxidative-stress-related heart diseases.

## 1. Introduction

Despite advances in disease prevention, diagnosis, and treatment, cardiovascular diseases (CVDs) are still in the leading position as the cause of mortality and morbidity worldwide. It is estimated that by 2030, nearly 23.6 million people will die from CVDs, primarily from heart disease and stroke, per year [1,2]. As both reactive oxygen species (ROS) production and microRNA (miRNA) expression signature have been associated with the development of CVDs, it is important to understand the crosstalk between ROS and miRNAs [3,4].

ROS are constantly released during mitochondrial oxygen consumption for energy production. Any imbalance between ROS production and its scavenger system induces oxidative stress. Oxidative stress, a critical contributor to tissue damage, is well-known to be associated with various diseases [5]. It has been long recognized that an increase of ROS can modify the cell-signaling proteins and has functional consequences, which mediate pathological processes included in the development of CVDs related to hypoxia, cardiotoxicity, and ischemia-reperfusion [6]. It was reported that myocardial ROS levels were elevated in animal models of ischemia/reperfusion injury [7] and heart failure [8]. Recent data from T. Wongsurawat study reveal elevated level of ROS species at the proteomic and transcriptomic level in the vessel wall [9,10,11].

MiRNAs are integrated into a group of small, naturally occurring and noncoding RNAs (size 21–25 nucleotides), which modulate gene expression at the post-transcriptional level. MiRNAs play a role as regulators of gene expression through binding to complementary sequences on the 3′-untranslated region (3′-UTR) of their target mRNA, thus inhibiting mRNA translation or promoting mRNA degradation [12,13]. Many miRNA genes are transcribed by enzyme RNA polymerase II from intergenic, intronic, or polycistronic loci as a long primary miRNA transcript (pri-miRNA), which is then cleaved by the enzyme Drosha endoribonuclease to a 70-nt-long hairpin structure with 2-nt-30 overhangs (pre-miRNA). Pre-miRNA is thereafter exported to the cytoplasm and processed by a second endoribonuclease enzyme (Dicer), to form a 22-nucleotide-long miRNA:miRNA* duplex with partial complementarity. One strand of this duplex then combines with the Argonaute (AGO) protein into the RNA-induced silencing complex (RISC), while the passenger strand gets degraded. One mRNA can contain multiple binding sites for different miRNAs, thus creating a complicated network of miRNA–mRNA interactions. MiRNAs are distributed in tissue-specific patterns and are able to regulate the expression of approximately 30% of human genes [14,15,16].

Numerous studies have shown that miRNAs have essential roles in cardiovascular development, pathology, regeneration, and repair and could be used for the diagnosis and prevention of cardiovascular diseases, such as hypertrophy, myocardial infarction, contractility defects, arrhythmias, etc. [3,17,18,19,20,21]. In response to increased ROS or stress stimuli, CVDs are obviously initiated and progressed by apoptosis, autophagy, necrosis, and fibrosis, as well as proliferation and migration of cardiomyocytes and endothelial cells, cardiac fibroblasts, and vascular smooth muscle cells. It was documented that miRNAs are involved in these processes [3,18,22]. Moreover, some miRNAs have been assigned as regulators of oxidative stress in the cardiovascular system by targeting ROS generators, antioxidant signaling pathways, and selected antioxidant effectors [23].

In this review, we tried to highlight recent findings about the association of miRNAs with the development of CVDs, including atherosclerosis, myocardial infarction, cardiac hypertrophy, or heart failure. There is evidence of interactions between cardiac miRNAs and ROS, but further studies need to be provided to reveal the molecular mechanisms of miRNAs regulating CVD diseases under ROS-related stress conditions.

## 2. Oxidative Stress and Cardiovascular System

The common pathological feature of most cardiac and vascular diseases is an imbalance of biological system in oxidation and antioxidation or between the generation and detoxification of ROS, which is generally called oxidative stress [24,25].

ROS are small reactive molecules implicated in the regulation of various cell functions and biological processes [26]. They are defined as molecules containing at least one atom of oxygen with higher reactivity than molecular oxygen, like superoxide anion (O_2_^−^), hydrogen peroxide (H_2_O_2_), hydroxyl radical (OH^▪^), peroxynitrite (ONOO^−^), hypochlorous acid (HOCl), and others [27]. In smaller or moderate concentrations, ROS can serve as signaling molecules, but an uncontrolled higher level of ROS leads to free-radical damage associated with the structural and functional alterations of proteins, lipids, and deoxyribonucleic acid [28,29].

The generation of ROS could be divided into two categories. Firstly, they are produced mainly by mitochondrial oxidative metabolism as a by-product or waste product, and secondly, as a cellular response to stress, xenobiotics, cytokines, and bacterial invasion, where ROS are formed intentionally as part of a signal transduction pathway [6,30]. Most relevant enzymatic sources of ROS are the nicotinamide adenine dinucleotide phosphate (NADPH) oxidases (NOXs), xanthine oxidase, uncoupled nitric oxide (NO) synthase, and mitochondria [24]. To the other incentives belong tumor necrosis factor-alpha (TNF-α), epidermal growth factor, Interleukin-1beta (IL-1β), hypoxia, and irradiation [6].

To ensure homeostasis in these processes, proteins with an antioxidant activity that protect an aerobic organism from increased toxicity of ROS have been found [31]. Major antioxidant proteins are superoxide dismutases (SODs), which catalyze conversion of superoxide into oxygen and hydrogen peroxide [32]. Hydrogen peroxide produced by SOD is then subsequently transformed into water and oxygen by other antioxidant proteins, glutathione peroxidases, catalases, and thioredoxins [33]. Taken together, to keep redox equilibrium is very difficult due to a variety of mechanisms, and, in the case of the antioxidant cell defense system that is suppressed by oxidative stress, many diseases can occur, especially in the cardiovascular system, where oxygen delivery to myocardium is approximately 1.6–1.8 times higher than in other tissues [34].

**Figure 1:**
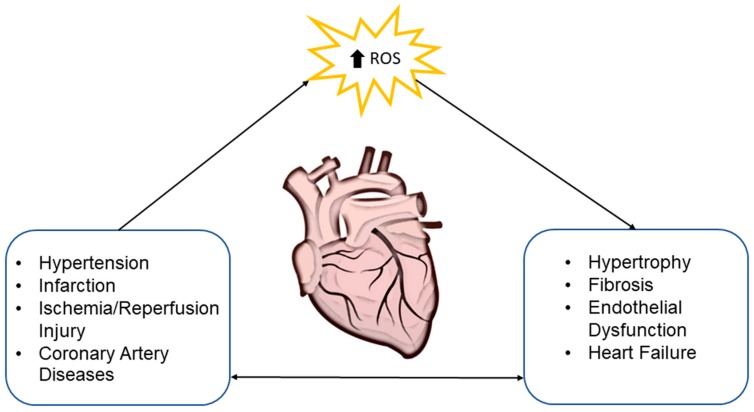
Impact of oxidative stress on the heart. Overproduction of reactive oxygen species (ROS) contributes to different cardiac pathologies, e.g., hypertrophy or fibrosis. These pathological changes in the heart may result in cardiovascular diseases, which may subsequently contribute to the production of ROS.

Increased levels of ROS are unfavorably implicated in the myocardial calcium handling, cardiac remodeling induced by hypertrophic signaling, apoptosis, and necrosis. Oxidative stress similarly has a negative effect on blood vessels, their function, angiogenesis, apoptosis, vascular tone, and genomic stability [24]. This suggests that cardiovascular risk factors with elevated ROS levels are interconnected with endothelial dysfunction. Dysregulated generation of ROS also contributes to the pathogenesis of atherosclerosis, heart failure, cardiomyopathy, and cardiac hypertrophy [35] (Figure 1).

It was observed that activation of neuroendocrine pathways, namely sympathetic and the renin-angiotensin-aldosterone system in patients with failing myocardium, was associated with oxidative stress [36,37]. In experimental and human studies dealing with heart failure, the elevated activity of NOX has been consistently observed [37,38]. For example, the implication of NOX isoforms in the development of left ventricular hypertrophy (LVH) was demonstrated in NOX2 knockout mice, where infusion of Angiotensin II resulted in less incidence of LVH compared to control wild-type mice infused with the same concentration of Angiotensin II induced LVH [39]. A similar conclusion was drawn from neonatal rat ventricular cardiomyocytes where activation of NOX2 was associated with angiotensin II-induced cardiac hypertrophy [40]. Besides that, inactivation of NOX2 leads to the reduction of infarct size in a model of myocardial infarction [41]. Activation of NOX4 also has an impact on heart failure [42]. This implication was shown in experimental study with transgenic mice with cardiac-specific overexpression of NOX4, where incidence of fibrosis, apoptosis, and enlargement of cardiomyocytes were found [43]. Besides, upregulated expression of NOX4 was associated with overexpression of lysocardiolipin acyltransferase-1, which was implicated in the catalysis of cardiolipin synthesis [44]. Upregulated lysocardiolipin acyltransferase-1 by oxidative stress damages phospholipid cardiolipin, a component of the inner mitochondrial membrane leading to increased levels of O_2_^−^, ONOO^−^, and NO radicals, which results in the decline of ATP production and disorganization of the dimeric ADP/ATP carrier functional capacity [45,46]. Unlike this, in mice with cardiac-specific deletion of NOX4 with applied transverse aortic constriction, the pathological changes were less pronounced [43]. Expression and activity of xanthine oxidase were also increased in patients with heart failure, whereas inhibition of this enzyme ameliorated heart contractility, as well as endothelial dysfunction [47].

Hypertension, hypercholesterolemia, hyperglycemia, and atherosclerosis are also typical with the activation of ROS enzyme sources [24]. Elevated ROS levels, activation of NOX [48], impairment of NO/cGMP signaling, and subsequently reduced acetylcholine-mediated vasodilation in hypertension models were induced by Angiotensin II stimulation [49]. Moreover, a decline in superoxide dismutase and glutathione peroxidase activity were inversely correlated with blood pressure in untreated hypertensive patients [50]. The process of atherosclerosis is closely linked with lack of NO production or its accelerated scavenging [51]. Generated ONOO^−^ induces transformation of smooth muscle cells into foam cells, as well as release of matrix metalloproteinases, which degrade atheromatous plaque and basement membrane of the endothelial cells leading to physical disruption of the plaques [26].

Another situation of oxidative-stress-related cardiac damage is heart transplantations, where the donor heart, graft, is exposed to cold ischemia-reperfusion injury associated with increased ROS. This leads to graft dysfunction, like allograft rejection, delayed graft function, or primary nonfunction, as well as to endothelial and parenchymal cell injury [52,53]. To increase success of the heart transplantation, experimental studies have focused on the enrichment of antioxidative substrates in cardioplegia solution and pump prime solution [54,55,56,57,58,59], suggesting the importance of continuous research into antioxidants.

## 3. Oxidative Stress and MiRNA

**Figure 2:**
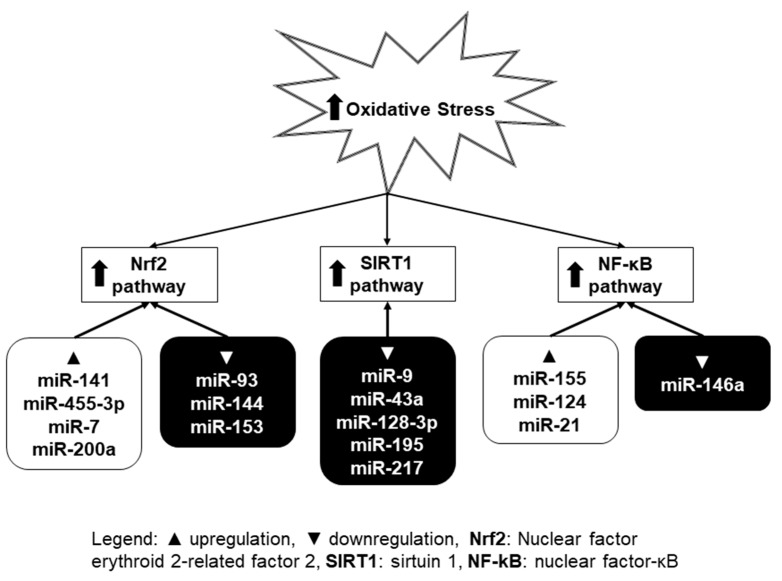
Selected signaling pathways (Nrf2, SIRT1, and NF-κB) influenced by miRNAs in situations with oxidative stress. ROS either inhibit or induce miRNA expression level. This leads to subsequent regulation of their target genes. Upper arrow represents increase of oxidative stress/activation of signaling pathway.

Many previous studies have demonstrated that multiple molecular mechanisms and signaling pathways can regulate oxidative stress [60]. Growing evidence has confirmed that miRNAs can be considered as potential targets and modulators of oxidative-stress-related pathways [61]. By analysis of miRNA expression signature implicated in oxidative stress-related pathways, several miRNAs were identified and termed as oxidative stress-responsive miRNAs [62]. The increasing number of studies shows that intracellular ROS can either inhibit or induce miRNA expression level, which results in subsequent biological effects through regulation of their direct target genes (Figure 2) [63]. Among them, several pathways (Nrf2—nuclear factor erythroid 2-related factor 2, SIRT1—sirtuin 1, and NF-κB—nuclear factor kappa B) have been the most intensively studied in connection with oxidative stress and miRNA. These will be further described in this section.

### 3.1. Nrf2 Pathway

Nrf2 plays a part in the cellular antioxidant defense system by upregulating the expression levels of antioxidant enzymes [64], such as glutathione S-transferase (GST), NAD(P)H:quinone oxidoreductase (NQO) 1, SOD1, and heme oxygenase (HO) 1, through binding to antioxidant response elements (AREs) in their promoters [23]. Under physiological conditions, Nrf2 is bound to its inhibitory protein, Kelch-like ECH-associated protein 1 (Keap1), which limits its transcriptional activity in the nucleus. Oxidative stress causes Nrf2 to dissociate from Keap1, which results in its binding to ARE and the transcription of downstream target genes [65].

Some miRNAs were reported to target Nrf2 directly. Zhu et al. revealed that miRNA-153 promotes oxidative stress by negatively regulating Nrf2 in an in vitro model of Parkinson’s disease [66]. The study of Sangokoya et al. shows that increased expression of miRNA-144 is associated with reduced Nrf2 levels in homozygous sickle cell disease (HbSS) reticulocytes and with decreased glutathione regeneration and attenuated antioxidant capacity in HbSS erythrocytes [67]. Another study found that the downregulation of miRNA-93 elevates Nrf2 expression and alleviates reactive oxygen species and cell apoptosis in diabetic retinopathy [68].

Besides directly targeting Nrf2, miRNAs may also target its regulators. Cheng et al. demonstrated that miRNA-141 attenuates UV-induced oxidative stress via targeting Keap1 to activate Nrf2 signaling in human retinal pigment epithelium cells and retinal ganglion cells [69]. MiRNA-7 represses Keap1 expression in human neuroblastoma cells, decreases the intracellular hydroperoxide level, and increases the level of the reduced form of glutathione [70]. Zhang et al. demonstrated that miRNA-455-3p activated the Nrf2/ARE signal pathway through suppressing Keap1, thereby suppressing oxidative stress and promoting osteoblasts growth [71]. Other authors showed that miRNA-200a controls Nrf2 activation by target Keap1 in hepatic stellate cell proliferation and fibrosis [72].

### 3.2. SIRT1 Pathway

The increasing number of studies confirms that SIRT1 is an important component of cellular responses to oxidative stress [73,74]. SIRT1 is a target of various redox-sensitive pathways [23]. To induce an antioxidant response, activated SIRT1 deacetylates multiple targets, including endothelial nitric oxide synthase (eNOS), peroxisome proliferator-activated receptor-γ coactivator 1-α (PGC1α), p53, Forkhead box O transcription factors (FoxO), Nrf2, and NF-κB [65]. In the cell, the deacetylation of FoxO1 by SIRT1 increases transcriptional activity and upregulates downstream antioxidants such as SOD2 and catalase [75].

Several miRNAs have been reported to influence oxidative stress by directly targeting SIRT1. Downregulation of SIRT1 by miRNA-34a promoted vascular smooth muscle cells senescence and inflammation in aged mouse aortas [76]. Similarly, miRNA-217 was identified as an endogenous SIRT1 inhibitor, which promotes endothelial senescence. MiRNA-217 was expressed in human atherosclerotic lesions and was negatively correlated with SIRT1 expression and with FoxO1 acetylation status [77]. Zhao el al. showed that miRNA-128-3p aggravated the doxorubicin-induced liver injury by promoting oxidative stress via targeting SIRT1 [78]. The study of Zhu et al. demonstrates a pro-apoptotic role of miRNA-195 in cardiomyocytes and identifies SIRT1 as a direct target of miRNA-195. The effect of miRNA-195 on apoptosis is mediated through the downregulation of SIRT1, Bcl-2 (B-cell lymphoma 2), and ROS production [79]. D′Adamo et al. identified miRNA-9 as a post-transcriptional regulator of SIRT1. MiRNA-9 and SIRT1 levels showed opposite changes in chondrocytes, following H_2_O_2_ treatment [80].

### 3.3. NF-κB Pathway

Excessive levels of ROS would also activate NF-κB pathway that is a redox-sensitive pathway [81]. NF-κB is present in all kinds of cells controlling the transcription of a wide variety of genes, including pro-apoptotic and pro-survival genes, pro-inflammatory cytokines, antioxidant and pro-oxidant enzymes, and many others [82]. The mammalian NF-κB family is composed of five members: p65 (RelA), RelB, c-Rel, NF-κB1 (p50 and its precursor p105), and NF-κB2 (p52 and its precursor p100), which can form homodimers and heterodimers among themselves. The NF-κB proteins are normally sequestered in the cytoplasm by a family of inhibitory proteins, including IκB family members. The most common activation reactions of NF-κB are represented by phosphorylation and activation of the IκB kinase complex [83].

One of the most important ways in which NF-κB activity influences ROS levels is via increased expression of antioxidant proteins such as SOD, glutathione peroxidase, or heme oxygenase. Since NF-κB is important in inflammation, some enzymes that promote the production of ROS (e.g., NOX2, inducible nitric oxide synthase (iNOS), cyclooxygenase (COX) 2, or cytochrome P450 enzymes) are also regulated as its targets, especially in cells of the immune system [82]. Abnormal NF-κB activity is frequently associated with an abnormal level of miRNAs, which is found to play critical roles in disease progression [84].

Downregulation of miRNA-155 ameliorates high-glucose-induced endothelial injury by inhibiting NF-κB activation and promoting HO-1 and NO production [71]. Gu revealed that miRNA-124 prevents H_2_O_2_-induced oxidative stress and apoptosis in human lens epithelial cells by suppressing the activation of the NF-κB pathway [85]. Results of Wei et al. indicated that NF-κB positively regulated miRNA-21 expression under oxidative stress, and programmed cell death protein 4 (PDCD4) was a direct target for miRNA-21 [86]. In another study, Xie et al. studied the role of miRNA-146a in the brain of chronic type 2 diabetes mellitus (cT2DM) rats. Increased inflammation and oxidative stress were associated with brain impairment in cT2DM rats, which were negatively correlated with miR-146a expression. The expressions of NF-κB p65 and its specific modulators were elevated in the brain of cT2DM rats, which might be inhibited by miR-146a [87].

Several other genes and related pathways have been described in the literature to be involved in the regulation of oxidative stress by miRNAs. Examples of these are MAPK (mitogen-activated protein kinase) signaling pathway, TGF-beta (transforming growth factor beta) signaling pathway, cell adhesion molecules (CAMs), calcium signaling pathway, VEGF (vascular endothelial growth factor) signaling pathway, etc. [61].

## 4. MiRNA in Oxidative-Stress-Induced Heart Diseases

Oxidative stress plays a crucial role in many cardiovascular diseases, like hypoxia, ischemia/reperfusion injury, or heart failure [88]. As mentioned before, intracellular ROS are formed in normal conditions as the result of normal mitochondrial respiration, but ROS are also produced during reperfusion in hypoxic tissue and in association with infection and inflammation, leading to pathological conditions of the heart [88,89]. One of the effects of ROS accumulation in cardiomyocytes is a different expression of noncoding RNAs (ncRNAs), subsequently contributing to cell apoptosis and heart pathology. Among these ncRNAs, miRNAs are the most intensively studied, as they have a huge impact on heart condition by inhibiting protein translation or target mRNA degradation [16,90,91].

### 4.1. Cardiac Hypertrophy

Oxidative stress generates many complex cellular changes in the heart which force it to adaptation in the form of cardiac hypertrophy of ventricles. These adaptations may provide initial salutary compensation to the arisen stress, sustained hypertrophic stimulation becomes maladaptive, worsening morbidity, and mortality risks because of congestive heart failure and sudden cardiac death [92]. During hypertrophy in different experiments with animal models or in clinical trials, changes in miRNA expression were observed—mainly miRNA-1 and -133. Zhao et al. demonstrated that their lower levels indicate a significant cardiac injury [93]. MiRNA-1 is connected with cardiomyocyte growth and hypertrophy most probably through the calcineurin/nuclear factor of activated T cell (NFAT) signaling pathway inhibition [94,95]. As a previous miRNA-1 case, also patients and animals with cardiac hypertrophy have lower levels of miRNA-133, probably by regulating antihypertrophic genes like guanosine diphosphate–guanosine triphosphate (GDP–GTP) exchange protein, or signal transduction kinase cell division control protein 42 (Cdc42) [96,97].

MiRNA-208 belongs to the cardiac-specific miRNAs, and its regulation is important in the processes of cardiac remodeling. Several studies demonstrated that cardiac hypertrophy is caused by switching of adult alpha myosin heavy chain (α-MHC, known as *Myh6*) to fetal beta myosin heavy chain (β-MHC, known as *Myh7*) gene expression. In experimental studies, deletion of miRNA-208a, which is encoded in the intron of the *Myh6* gene, leads to the decreased expression of the *Myh7* gene in response to stress and to hypertrophy [88,98]. These results were confirmed in the study of Rawal et al., where inhibition of miRNA-208a hampers the activation of β-MHC and hypertrophic response [99].

Important miRNAs involved in the cardiac hypertrophy are also miRNA-22 (influence phosphatidylinositol-3-kinase (PI3K)-protein kinase B (AKT)) [100], miRNA-212/132 family (active through antihypertrophic FoxO3 transcription factor), or miRNA-199 (miRNA-199a targets the pro-autophagic and antihypertrophic factor glycogen synthase kinase 3β; miRNA-199b acts through targeting tyrosine phosphorylation regulated kinase 1A (*Dyrk1a*) gene, involved in the phosphorylation of NFAT factors) [101,102,103]. Other studies dealing with miRNAs associated with cardiac hypertrophy observed changed expression of miRNA-21, -18b, -195, -199, -29, -22, or -23 levels [104,105,106,107].

### 4.2. Ischemia/Reperfusion Injury

Cardiac ischemia/reperfusion (I/R) injury involves the damage caused by reduced coronary blood flow, causing depletion of ATP, reduced partial pressure of oxygen, and production of toxins. Reperfusion leads to further damage through generation of ROS and a proton gradient across both the sarcolemma and the inner mitochondrial membrane [108]. Many miRNAs are involved in these processes, either as a result of damage caused by ROS generation or directly responsive to ROS.

One of the most promising miRNAs for potential use as a diagnostic or therapeutic target is miRNA-24-3p. In a very recent study of Xiao et al., decrease expression of miRNA-24-3p during induced ischemia/reperfusion injury in mouse hearts and decreasing levels of apoptosis of cardiomyocytes caused by ROS during ischemia/reperfusion injury after application of miRNA mimics were confirmed [109]. In addition to these observations, the authors identified the Keap1-Nrf2 pathway as one of the possible targets of miRNA-24-3p [109].

Lusha et al. showed that miRNA-144, which is primarily connected with the regulation of apoptosis in human cancer diseases, is another miRNA with changed expression levels in the I/R model through regulation of FoxO1. The authors observed in their study reduced infarct size and apoptosis in cardiomyocytes during the upregulation of miRNA-144 and increased sensitivity to I/R in the situation with depleted miRNA-144 [110]. FoxO1 protein is an important transcription factor which mediates apoptosis by activating iNOS expression in cardiomyocytes [111], and it can be regulated by sirtuin 1 in the cardiovascular system [112].

In another study, Fang and Yeh explored the role of miRNAs in cardiomyocyte apoptosis induced by ischemia. They found an increased level of miRNA-302 expression induced by hypoxia/reoxygenation injury. This aggravated cardiomyocyte apoptosis probably by inhibiting antiapoptotic protein myeloid cell leukemia 1 (Mcl-1) expression, thereby activating pro-apoptotic molecules. Based on this data, the authors suggested that elevated miRNA-302 levels can be detrimental to cells, but decreased levels are beneficial and can lead to effective therapeutic intervention [113]. Several publications reveal profound effects of myocardial ischemia on miRNA transcript in the vessel wall and vascular smooth muscle cells, in particular [114,115,116].

MiRNA-23a promotes cardiomyocyte apoptosis and myocardial infarction induced by I/R through directly suppressing the expression of manganese SOD, an important antioxidant for scavenging of superoxide [117]. This is one of the endogenous enzymes that protects cells from oxidative stress. Wang et al. observed that miRNA-1 worsens cardiac oxidative stress by post-transcriptional modification of the antioxidant network in the I/R injury C57BL/6 mice model. They found that miRNA-1 reduced the protein levels of antioxidant enzymes glutamate cysteine ligase (Gclc), SOD1, and glucose-6-phosphate dehydrogenase (G6PD) under oxidative stress conditions [118]. Other miRNAs, such as miRNA-130a [119] and miRNA-98 [120], are investigated to be associated with ROS-related cardiomyocyte apoptosis. MiRNA-208a promoted apoptosis and oxidative stress in the I/R injury rat model by regulation of protein tyrosine phosphatase receptor type G and protein tyrosine phosphatase, non-receptor type 4 [121].

Another situation of ischemia/reperfusion injury is heart transplantations. During the transplantation process in the final step, where heart graft is connected to the circulatory system of the recipient and when reperfusion of the graft is started, there is an excessive production of free radicals, what could lead to graft failures and lower long-term survival rate of the patients [122]. In several works, more attention is focused on miRNA′s changed expression after transplantation, making miRNAs as an ideal candidate for biomarkers of transplant rejection [122,123,124]. Zhou et al. found upregulation of miRNA-711, -2137, -705, -5130, -346, -714, and -744 and downregulation of miRNA-210, -490, -491, -425, -423-3p, and -532-3p in experiments on C56BL/6 mouse animal models after heart transplantation in I/R injured hearts [122].

MiRNAs were measured in the samples from endomyocardial biopsies (EMB) and blood serum in 30 patients with rejecting heart allograft and 30 patients without allograft rejection. MiRNA analyzes revealed changed expression of miRNA-10a, -31, -92a, and -155 in EMB, as well as in blood serum [125]. These miRNAs are strongly associated with inflammatory processes, as they influenced NF-κB, TNF-α, interleukins -6, -8, and -1, monocyte chemoattractant protein-1, eNOS, or vascular cell adhesion protein [126,127,128]. Wei et al. also suggested that miRNA-183, -182, and -96 have an important function in the regulation of graft rejection probably through regulation of FoxO1 expression and could be new potential noninvasive biomarkers of allograft rejection in heart transplantation [129].

All these results suggested the potential role of miRNAs in the regulation and adaptation of transplanted allografts and their potential use as biomarkers in grafts rejection.

### 4.3. Coronary Artery Diseases (CAD)

CAD is an atherosclerotic disease which is inflammatory in nature. Atherosclerosis starts due to the accumulation of lipoproteins in the intima of the coronary vessels. Oxidized or modified low-density lipoprotein then attract leukocytes into the intima of the coronary vessels, which can be scavenged by macrophages, leading to the formation of foamy cells. The atherosclerotic plaque starts developing. Cell death or apoptosis occurs commonly in the atherosclerotic lesions. The modified lipoproteins propagate inflammatory responses. As a result, obstruction of blood flow occurs, and this leads to a mismatch between myocardial oxygen demand and supply [130].

Endothelial cell apoptosis under oxidative stress plays a critical role in the initiation and progression of atherosclerosis [131,132,133,134,135,136,137,138,139,140,141]. Li et al. observed that overexpression of miRNA-210 caused inhibition of apoptosis and reduction of ROS level in human umbilical vein endothelial cells (HUVECs) treated with H_2_O_2_ and also downregulation of caspase levels. This study leads to the conclusion that miRNA-210 could have a role in the protection against oxidative-stress-induced apoptosis in HUVECs [131].

MiRNA-24 is highly expressed in the vessel wall and changes of its expression are connected with dysfunction and injury of vascular endothelial cells [132]. It is believed that miRNA-24 participates in many pathophysiological processes including I/R injury or vascular oxidative stress [134,135,136,137]. In an experimental study by Zhang et al., miRNA-24 was upregulated, and it has a supportive effect on vascular endothelium repair by attenuating oxidative-stress-induced damage of endothelial cells. In this case, miRNA-24 regulated the Nrf2/HO-1 signaling pathway indirectly by regulating of Keap1 after influencing of O-linked β-N-acetylglucosamine transferase gene (*Ogt*). On the other hand, authors confirmed that miRNA-24 affects the expression of SOD, malondialdehyde, and glutathione peroxidase [137].

MiRNA-92a overexpression impairs endothelial function and suppresses HO-1 expression in endothelial cells. Inhibition of miRNA-92a attenuates oxidative stress and improves endothelial function through enhancing HO-1 expression and activity in diabetic mouse aortas [138]. Yamac et al. observed markedly lowered expression of miRNA-199a in patients with coronary artery disease [139]. In parallel, they also showed the induction of cardioprotective protein SIRT1, a potential target of miRNA-199. Significantly increased expression of miRNA-146a was observed in patients with acute coronary syndrome. This miRNA is connected with the inflammatory pathway by regulating NF-κB [140]. According to O´Sullivan et al., miRNA-93-5p belongs to one of the strongest predictors of coronary artery disease when its expression was significantly upregulated in the patients with CAD, most likely through modulation of ATP-binding cassette A1 (*ABCA1*) gene [141]. ABCA1 plays an important role in cholesterol homeostasis and atherogenesis, and it could be reduced by oxidative stress [133].

### 4.4. Heart Failure

A complex syndrome resulting from structural or functional cardiac disorders, leading to disability of ventricle to fill or eject blood, is called heart failure [142,143,144], and it is considered to be one of the leading cause of morbidity and mortality worldwide [142,145,146,147,148]. Development of heart failure depends on many circumstances in the organism, but one of the key pathophysiological pathways for it is oxidative stress [149,150,151]. According to a lot of animal and human studies, multiple miRNAs are changed in models of heart failure, including miRNA-199b, -195, -100, -133, -24, and -208 [94,152,153,154,155,156]. Upregulated levels of miRNA-199b were measured during heart failure and appeared to target the calcineurin/NFAT pathway. It was proved that this calcineurin/NFAT pathway is activated after oxidative stress stimuli [157,158]. In the in vivo experiments, inhibition of miRNA-199b caused normalization of the expression of Dyrk1a, a reduction of nuclear NFAT activity, and inhibition of hypertrophy and fibrosis in mouse models of heart failure [101,159]. Changed expressions of miRNA-1, -214, -29b, -342, -7, -107, -126, -125, -122, -423-5p, -320a, -650, -1228, -662, -583, -3175, -21, -22, and -92b have been shown in other studies of heart failure [106,152,156,160,161,162,163].

A brief review of selected miRNAs included in the cardiovascular diseases caused by oxidative stress is provided in Table 1.

## 5. Future Perspectives of Using MiRNA in Disease Diagnosis and Treatment

Since 2001, miRNAs have been recognized as biomarkers and possible therapeutic targets for the diagnosis and treatment of diseases [165]. One of the biggest advantages for using miRNAs as biomarkers is their stability under many different conditions. MiRNAs can be stored at room temperature, frozen, or thawed [166]. Bioavailability of miRNA is another great advantage. MiRNAs can be isolated from various biological materials, like from peripheral blood, fresh and frozen tissues, or formalin-fixed, paraffin-wax-embedded samples, but also from saliva, epithelium of the skin, or hair [167,168]. Difficulties in the use of therapeutically altering miRNAs lie in their non-specificity— single miRNA can target many genes and influence more than one gene expression, so they could affect also other pathways in the organisms [169]. MiRNAs impose a relatively modest effect on their target, reflecting that individual mRNAs are targeted by multiple miRNAs, while the cellular proteome might be able to compensate the absence of a single miRNA [170].

Manipulation of RNA using miRNA mimics and antagomirs holds significant therapeutic potential for treating a variety of diseases. With recent technological advances, identification and validation of potential therapeutic miRNA targets are readily available [165]. Treatment of diseases by modulation of selected miRNAs in the organisms is based on two approaches. First, miRNA mimics is an approach for gene silencing due to generating synthetized artificial double-stranded miRNA-like RNA fragments. These molecules are able to bind to target mRNA and suppressed genes [171]. The second approach uses antagomirs, chemically designed oligonucleotides. These oligonucleotides specifically inhibit target miRNA by binding to them, which leads to reduction of RISC activation and to upregulation of genes [172,173]. MiRNAs could be modulated also by miRNA sponges (target mimicry), masking, and erasers. MiRNA sponges contain a binding site for the miRNA family, leading to the blocking of the activity of miRNAs [174,175]. Masking is based on the occupation of the binding site on target mRNA by oligonucleotides [176]. Erasers are oligonucleotides complementary to specific miRNA, leading to inhibition of its function [177]. However, delivery of anti-miRNAs and miRNAs in vivo may prove to be challenging [165].

## 6. Conclusions

Oxidative stress is one of the important contributing factors in cardiovascular disease genesis and development. Excessive ROS production has a significant impact on the pathogenesis of cardiovascular diseases related to atherosclerosis, cardiomyopathy, ischemia/reperfusion, and heart failure. Published literature highlights the increasing importance of studying the role of redox-sensitive miRNAs to identify more effective biomarkers and develop better therapeutic targets for oxidative-stress-related diseases. It is necessary to define the roles of individual miRNAs and their important targets, to determine their potential for possible diagnosis/treatment of cardiovascular disorders. Although a number of targets of oxidative-stress-responsive miRNAs have been identified, e.g., Nrf2, SIRT1, and NF-κB, future studies are still needed to determine further potential targets and their links to cardiovascular disease. MiRNA may be a promising novel tool and means in the clinical diagnosis, prognostic evaluation, and even therapeutic intervention of oxidative-stress-related CVD. The knowledge of the crosstalk between miRNAs, ROS, and cardiovascular diseases can contribute to new therapeutic approaches based on the suppression of ROS effects, with the potential to ameliorate or prevent the progression of cardiovascular diseases. However, several studies are still required to validate the present findings before the application of miRNA in clinical practice.

## Figures and Tables

**Table 1:** List of selected miRNAs with function in oxidative-stress-induced cardiovascular diseases.

Disease	miRNA	Expression	Target	References
**Cardiac hypertrophy**	miRNA-1	Downregulated	Mef2a; Gata4	[93,94,95]
	miRNA-133	Downregulated	GDP–GTP exchange protein; Cdc42	[93,96,97]
	miRNA-208a	Downregulated	*Myh7*	[18,98,99]
**Ischemia/reperfusion injury**	miRNA-24-3p	Downregulated	Keap1/Nrf2	[109]
	miRNA-144	Downregulated	FoxO1	[110,111]
	miRNA-302	Upregulated	Mcl-1	[113]
	miRNA-23a	Upregulated	MnSOD	[117]
**Heart transplantation**	miRNA-10a	Downregulated	NF-κB	[126]
	miRNA-31	Upregulated	TNF-α	[127]
	miRNA-92a	Upregulated	Integrin a5, S1P1, MKK4, eNOS	[128]
	miRNA-155	Upregulated	T-cell receptor, IFN receptor	[164]
**Coronary artery diseases**	miRNA-24	Downregulated	*Ogt*, Keap1/Nrf2	[132,134,135,136,137]
	miRNA-92a	Upregulated	HO-1	[138]
	miRNA-199a	Downregulated	SIRT1	[139]
**Heart failure**	miRNA-199b	Upregulated	calcineurin/NFAT	[101,157,158,159]
	miRNA-21	Upregulated	natriuretic peptide B	[156,163]

Mef2a—Myocyte-specific enhancer factor 2A, Gata4—Transcription factor GATA-4, GDP–GTP exchange protein–guanosine diphosphate–guanosine triphosphate exchange protein, Cdc42—Cell division control protein 42, *Myh7*—beta myosin heavy chain, Keap1/Nrf2—Kelch-like ECH-associated protein 1/Nuclear factor erythroid 2-related factor 2, FoxO1–Forkhead box protein O1, Mcl-1—Myeloid cell leukemia 1, MnSOD—manganese-dependent superoxide dismutase, NF-κB–nuclear factor kappa B, TNF-α–tumor necrosis factor alpha, S1P1–Sphingosine-1-phosphate receptor 1, MKK4—Mitogen-activated protein kinase kinase 4, eNOS—Endothelial nitric oxide synthase, INF receptor–interferon receptor, *Ogt*—O-linked β-N-acetylglucosamine transferase, HO-1—Heme oxygenase 1, SIRT1—sirtuin 1, NFAT—Nuclear factor of activated T cells.
